# Cytokine release syndrome was an independent risk factor associated with hypoalbuminemia for patients with relapsed/refractory hematological malignancies after CAR-T cell therapy

**DOI:** 10.1186/s12885-023-11540-8

**Published:** 2023-11-02

**Authors:** Shuyi Ding, Rongrong Chen, Linqin Wang, Cheng Zu, Xiaoyu Zhou, Jianli Zhang, Mingming Zhang, Aiyun Jin, Tingting Wang, Yongxian Hu

**Affiliations:** 1 Department of nursing, The First Affiliated Hospital, Zhejiang University School of Medicine, Hangzhou, China; 2 Bone Marrow Transplantation Center, The First Affiliated Hospital, Zhejiang University School of Medicine, No.79, Qingchun Road, Hangzhou, China; 3 Institute of Hematology, Zhejiang University, Hangzhou, China; 4 Liangzhu Laboratory, Zhejiang University Medical Center, 1369 West Wenyi Road, Hangzhou, 311121 China; 5 Department of Nutrition, The First Affiliated Hospital, School of Medicine, Zhejiang University, Hangzhou, China

**Keywords:** Chimeric Antigen Receptor (CAR)-T cell therapy, Cytokine release syndrome, Hypoalbuminemia, Hematological malignancies

## Abstract

**Background & aims:**

This study aims to assess the nutritional status of patients during the different phases of the Chimeric Antigen Receptor (CAR)-T cell therapy and to identify prominent risk factors of hypoalbuminemia in patients after CAR-T treatment. The clinical consequences of malnutrition in cancer patients have been highlighted by growing evidence from previous clinical studies. Given CAR-T cell therapy's treatment intensity and possible side effects, it is important to provide patients with sufficient medical attention and support for their nutritional well-being.

**Methods:**

This study was conducted from May 2021 to December 2021 among patients undergoing CAR-T cell therapy at the Bone Marrow Transplantation Center in The First Affiliated Hospital of Zhejiang University School of Medicine. Logistic regression analysis was performed to investigate the risk factors associated with hypoalbuminemia. Participants were divided into the cytokine release syndrome (CRS) group (*n* = 60) and the non-CRS group (*n* = 11) to further analyze the relationship between hypoalbuminemia and CRS.

**Results:**

CRS (OR = 13.618; 95% CI = 1.499–123.709;* P* = 0.013) and baseline albumin (ALB) (OR = 0.854; 95% CI = 0.754–0.967; *P* = 0.020) were identified as the independent clinical factors associated with post-CAR-T hypoalbuminemia. According to the nadir of serum albumin, hypoalbuminemia occurred most frequently in patients with severe CRS (78.57%). The nadir of serum albumin (*r* =  − 0.587, *P* < 0.001) and serum albumin at discharge (*r* =  − 0.315, *P* = 0.01) were negatively correlated for the duration of CRS. Furthermore, patients with hypoalbuminemia deserved longer hospitalization (*P* = 0.04).

**Conclusions:**

CRS was identified as a significant risk factor associated with post-CAR-T hypoalbuminemia. An obvious decline in serum albumin was observed as the grade and duration of CRS increase. However, further research is still needed to elucidate the mechanisms of CRS-associated hypoalbuminemia.

**Supplementary Information:**

The online version contains supplementary material available at 10.1186/s12885-023-11540-8.

## Introduction

Chimeric antigen receptor (CAR)-T cell therapy is a novel and adoptive immunotherapy that revolutionized the treatment of hematological malignancies, achieving 81% [1], 52–67% [2] and 73–85% [3] complete response (CR) rates in patients with refractory relapsed acute B-cell leukemia, B-cell lymphoma, and multiple myeloma, respectively, in different clinical trials. Cancer patients treated with CAR-T experienced symptoms ranging from mild constitutional symptoms such as fever, anorexia, malaise, headache, vomit, and diarrhea to severe symptoms such as life-threatening hemodynamic instability and multi-organ failure. These symptoms could potentially lead to malnutrition.

The aetiologia of nutritional issues in oncological patients is complex, which is primarily linked to the impairment of anti-cancer therapy and the metabolic disorders of the tumor itself [4]. The prevalence of malnutrition varies from 14.5% to 36.8% in hematological malignancy through different assessment tools [5–7]. It is well known that malnutrition impacts curative effects and increases the risk of morbidity and mortality [8], leading to poor clinical and economic consequences [9, 10]. The main characteristics of malnutrition are loss of weight and skeletal muscle. Malnutrition is closely related to sarcopenia, which leads to decreased lean body mass and muscle performance [11]. While many well-established factors contribute to malnutrition (e.g., Digestive disorders, cachexia), they have not been fully evaluated in the context of CAR-T cell therapy. Malnutrition should be considered a changeable status. Moreover, the timing of the nutritional interventions is essential.

Herein, we periodically assess the nutritional status of patients during the different phases of the CAR-T treatment. A clinical data-based prospective study was performed to identify the risk factors of post-CAR-T malnutrition and to further clarify its relationship with CRS, drawing some practical conclusions to support the multidisciplinary management of malnutrition.

## Methods

### Study design

From May 2021 to December 2021, 71 patients after CAR-T cell therapy were enrolled in this clinical trial (ChiCTR2100046474) conducted at the Bone Marrow Transplantation Center in The First Affiliated Hospital of Zhejiang University School of Medicine. All admitted patients were evaluated at the time of admission, referring to NRS 2002 Nutrition Risk Screening Form. This clinical trial was designed to explore nutritional status during CAR-T treatment and evaluate the risk factors of post-CAR-T hypoproteinemia and its correlation with CRS. The inclusion criteria were as follows: 1) Age ≥ 13 and < 70 years; 2) Expected survival time is more than 3 months; 3) Patients provided informed consent. The exclusion criteria were as follows: 1) Women who are pregnant or breastfeeding; 2) Patients had other uncontrolled medical conditions that the investigator deemed unsuitable for enrollment: 3) Patients with incomplete data.

All participants provided written informed consent. The patients were divided into the CRS group (*n *= 60) and the non-CRS group (*n* = 11). The treatment protocol included a lymphodepletion regimen consisting of 2 days of cyclophosphamide 0.5 g/m^2^ and 3 days of fludarabine 30 mg/m^2^/d, as well as CAR-T cell infusion. Patients with CRS received supportive treatment with tocilizumab (humanized monoclonal antibody against the IL-6 receptor) or corticosteroids. Ethical approval was obtained from the Ethics Committee of the First Affiliated Hospital of Zhejiang University School of Medicine. The study was registered at the Chinese Clinical Trial Registry (ChiCTR2100046474).

### Data collection and assessment

Data on blood nutrition, clinical baseline, CRS-related indicators, and severity were collected for all patients treated with CAR-T cells at our center. The primary outcomes were nutrition-related indicators and the incidence of hypoalbuminemia. In order to observe the dynamic changes in the nutritional status of patients during the CAR-T cell therapy, nutrition-related indicators at different time points were recorded, including serum albumin, total protein, triglyceride, total cholesterol, body mass index (BMI), grip strength, upper arm length, mid-calf circumference (MAC), and triceps skinfold thickness (TSF). CRS peak was defined as the day with the highest or most frequent fever. The prerequisite for CRS diagnosis is fever (temperature ≥ 38 °C which is not attributable to any other cause, within 24 h to 3 weeks after CAR-T cell administration). The diagnosis should also be combined with Hypotension, Hypoxia, and other systemic symptoms, according to the American Society for Transplantation and Cellular Therapy (ASTCT) Consensus Grading for Cytokine Release Syndrome (see supplementary material for detail) [12]. The severity of CRS could be classified into no CRS (grade 0), mild CRS (grade 1 or grade 2 CRS), and severe CRS (grade 3 or grade 4) subcategories. Serum albumin < 35 g/L is identified as hypoalbuminemia.

### Statistical analysis

SPSS 22.0 software (version 22.0; IBM, Armonk, New York) was used for statistical analysis. Biorender was used to draw the graphs. Mann–Whitney U test was utilized in the comparison of measurement data between the two groups. Spearman’s correlation coefficient was used to analyze the correlation between the duration of CRS and length of hospital stay with serum albumin. The correlation between the patients’ clinical parameters and hypoalbuminemia was assessed using univariate and multivariate logistic regression analysis. *P* < 0.05 was considered significant.

## Results

### Study cases and baseline characteristics

A total of 71 patients after CAR-T cell therapy (41males and 30 females) were enrolled, with a median age of 55 years (range, 17 to 69 years) and a median BMI of 22.08 kg/m^2^ (range, 2.73 to 32.15). Among the enrolled patients, 35 (49.3%) were diagnosed with acute leukemia, 17(23.9%) patients with lymphoma, and 19(26.8%) with multiple myeloma. The median chemotherapy cycle of the cases was 7. Nine patients undergone auto-HSCT and 3 patients receive allo-HSCT. During the study period, 60 patients developed CRS and 11 patients did not. Detailed baseline characteristics are shown in Table 1.

**Table 1:** Baseline characteristics of patients

**Characteristics**	Total (*n* = 71)
Age, years, median (range)	55 (17–69)
Male, n (%)	41 (57.75)
BMI, kg/m^2^, median (range)	22.08 (2.73–32.15)
Residence, n (%)	
Urban	44 (61.97)
Rural	27 (38.03)
Educational level, n (%)	
Primary education or less	22 (30.99)
Secondary school	38 (53.52)
University education	11 (15.49)
Diagnosis, n (%)	
Acute leukemia	35 (49.30)
Lymphoma	17 (23.94)
Multiple myeloma	19 (26.76)
Marital status, n (%)	
Married	64 (90.14)
Unmarried or divorced	7 (9.85)
Occupation, n (%)	
Retiree	15 (21.13)
Mental laborer	17 (23.94)
Manual laborer	39 (54.93)
Principal caregiver, n (%)	
Parents	8 (11.27)
Spouse	60 (84.51)
Offspring or thers	3 (4.23)
History of HSCT, n (%)	
Yes	12 (16.90)
No	59 (83.10)
Smoking history, n (%)	
Yes	10 (14.08)
No	61 (85.92)
Drinking history, n (%)	
Yes	6 (8.45)
No	65 (91.5)
Diabetes, n (%)	
Yes	9 (12.68)
No	62 (87.32)
Hypertension, n (%)	
Yes	10 (14.08)
No	61 (85.92)
HBV infection, n (%)	
Yes	7 (9.85)
No	64 (90.14)
Baseline LDH, U/L, median (range)	232.50 (46.00–1995.00)
Baseline ALB, g/L, median (range)	42.30 (28.90–53.60)
Baseline TP, g/L, median (range)	65.30 (49.50–101.50)
Baseline TG, mmol/L, median (range)	1.84 (0.56–8.55)
Baseline TC, mmol/L, median (range)	4.29 (1.43–7.59)
Hospital stay, days, median (range)	22 (9–80)

### The nutritional change among CRS and non-CRS groups

**Fig. 1:**
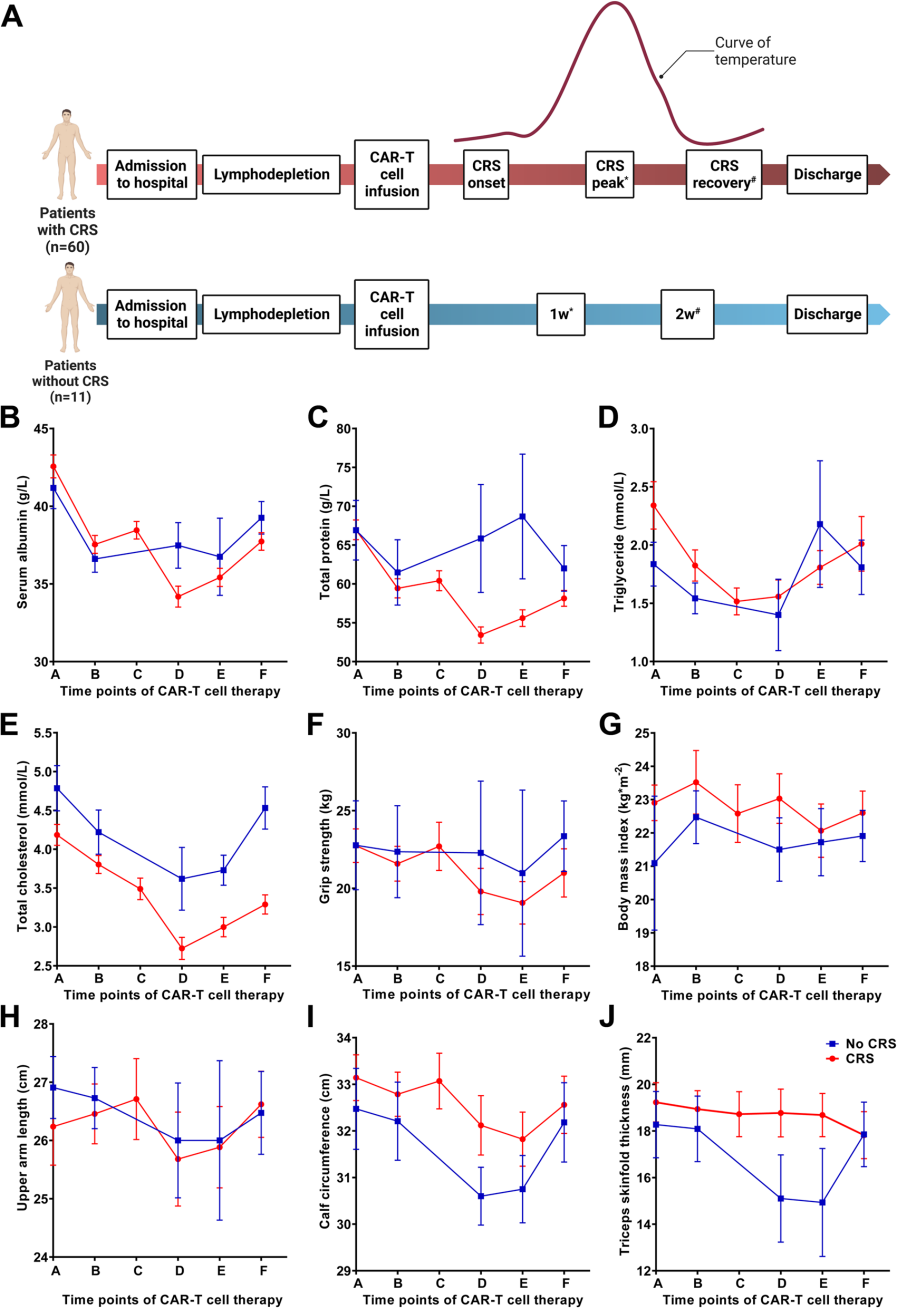
Overview of nutritional change among CRS and non-CRS groups. * “CRS peak” among patients with CRS and “1 week after CAR-T cell infusion” among patients without CRS were noted with time point “**D**”. # “CRS recovery” among patients with CRS and “2 weeks after CAR-T cell infusion” among patients without CRS were noted with time point “**E**”. * # Among patients without CRS, the nutritional data of “1 week or 2 weeks after CAR-T cell infusion” might be not available if they were discharged from the hospital early

The nutritional status of all patients was assessed when they were admitted to the hospital, during CAR-T cell infusion treatment, and discharged. For patients with CRS, nutrition-related indicators at CRS onset, peak, and recovery stages were recorded. For patients without CRS, these indicators were monitored at 1 week and 2 weeks after CAR-T cell infusion (Fig. 1A). Serum albumin and total protein showed a significant downward trend at the beginning of the CRS and reached their lowest value at CRS peak, which was not obvious in the non-CRS group (Fig. 1B, C). Triglyceride level, total cholesterol level, upper arm length, calf circumference, and Triceps skinfold thickness decreased obviously after CAR-T cell infusion with or without CRS, while BMI and grip strength remained stable during treatment (Fig. 1D, E, F, G, H, I, J).

### Univariate and multivariable logistic regression analysis of patients’ clinical parameters associated with post-CAR-T hypoalbuminemia

To determine risk predictors for post-CAR-T hypoalbuminemia, multivariate logistic regression was applied to the three significant variables identified by univariate analysis (*P* < 0.1). The three significant variables are CRS (*P* = 0.014), baseline ALB (*P* = 0.022) and baseline LDH (*P* = 0.082). Finally, CRS (odds ratio (OR) = 13.618, 95% confidence interval (CI) 1.499–123.709;* P* = 0.013) and baseline ALB (OR = 0.854, 95% CI 0.754–0.967; *P* = 0.020) were identified as the independent factors associated with post-CAR-T hypoalbuminemia.

### Further analysis of the correlation between hypoalbuminemia and the severity and duration of CRS

**Fig. 2:**
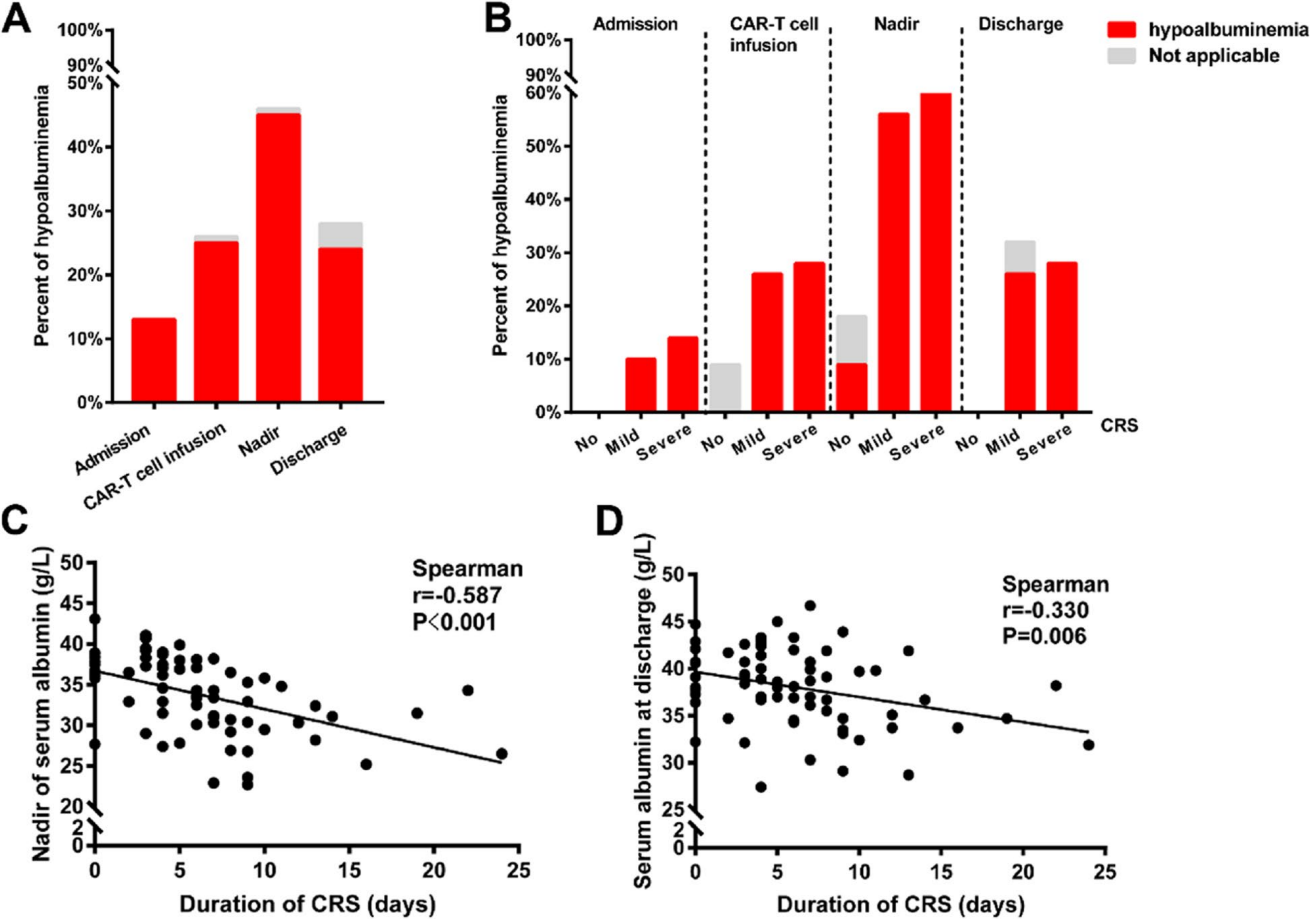
The incidence of hypoalbuminemia is associated with the severity and duration of CRS

The incidence of hypoalbuminemia events varied based on the level of albumin measured at different stages of hospitalization (Fig. 2A). The incidence of hypoalbuminemia was lowest at admission (12.68%) and was highest at the nadir of serum albumin (45.07%). The prevalence of hypoalbuminemia was 23.94% (42/117) at discharge. The severity of CRS could be divided into non-CRS (grade 0), mild CRS (grade 1 or grade 2), and severe CRS (grade 3 or grade 4) subcategories. Patients with severe CRS tend to have a higher probability of occurrence of hypoalbuminemia. According to the nadir of serum albumin, hypoalbuminemia occurred most frequently in patients with severe CRS (78.57%), the incidence of which was slightly lower at 56.52% in the mild CRS group (Fig. 2B).

**Fig. 3:**
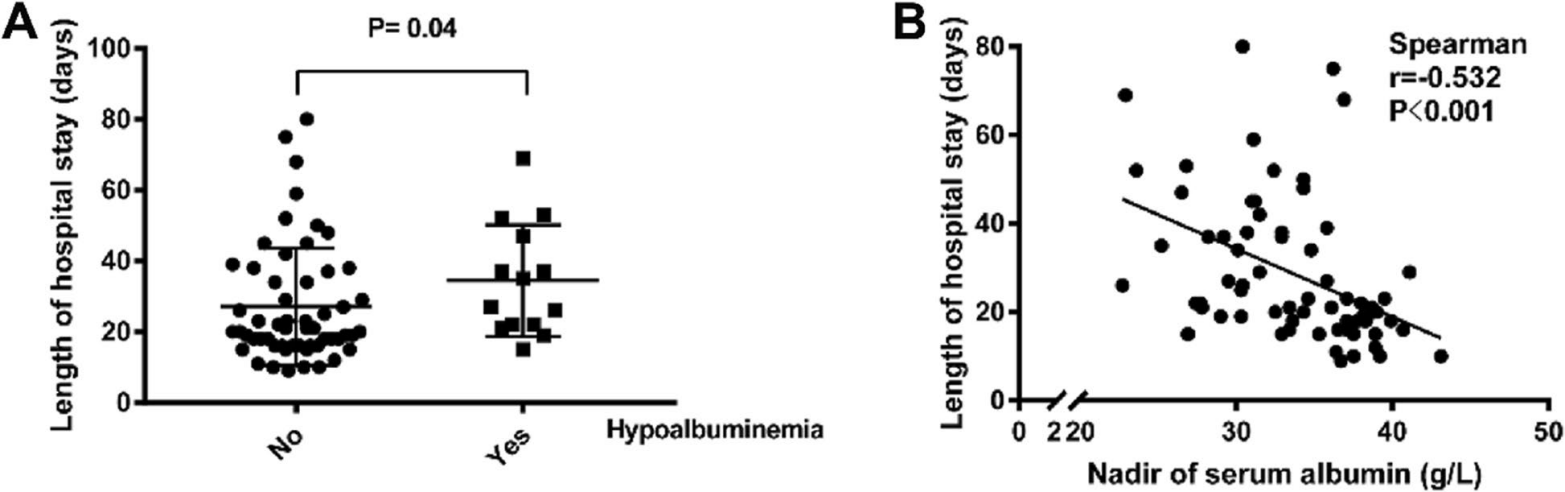
Hypoalbuminemia is associated with prolonged hospital stays

Spearman correlation analysis showed that nadir of serum albumin (*r* =  − 0.587, *P* < 0.001) and serum albumin at discharge (*r* =  − 0.315, *P* = 0.01) were negatively correlated duration of CRS (Fig. 2C, D). Patients with hypoalbuminemia had significantly longer hospital stays than patients without (*P* = 0.04) (Fig. 3A). And the nadir of serum album was also negatively associated with prolonged hospital stay (*r* =  − 0.532, *P* < 0.001) (Fig. 3B).

## Discussion

The nutritional status of patients treated with CAR-T cell therapy, which may cause both clinical and economic consequences, has been rarely studied. Indeed, this study is the first to assess changes in nutritional status and evaluate the risk factors of malnutrition in patients with hematological malignancies after CAR-T cell therapy. The change in nutritional status was presented in groups with CRS and without CRS during the different phases of CAR-T treatment. There is considerable biological plausibility for a marked decline of nutrition-related indicators after CAR-T cell infusion, including triglyceride, total cholesterol, upper arm length, and calf circumference, which may be primarily attributed to the treatment-related nutrition side effects caused by lymphodepletion regimen and CAR-T cell therapy.

The results of this study highlight the tendency for serum albumin to decline during hospitalization is particularly significant in patients with CRS. The multivariate analysis has confirmed that CRS is an independent risk factor of post-CAR-T hypoalbuminemia. Meanwhile, our previous retrospective study has also found that the albumin level and total cholesterol level after CAR-T cell infusion was negatively correlated with CRS grades [13]. Therefore, we reasonably hypothesized that a unique mechanism exists wherein CRS contributes to post-CAR-T hypoalbuminemia.

One possible explanation lies in the fact that an inflammatory state may lead to albumin depletion. Both levels of serum albumin and total protein reached their lowest values at the CRS peak. The growing evidence showed that T cell metabolism is strictly linked to nutritional status [14, 15]. The pro-inflammatory cytokines (e.g., interleukin 1 (IL-1), IL-6, tumor necrosis factor- α (TNF- α)) disrupt the metabolism of carbohydrates, fats, and proteins throughout the body [16–19].

The post-CAR-T hypoalbuminemia may also attribute to increased consumption and decreased gastrointestinal intake caused by inflammation. The levels of serum albumin changed at different treatment stages. For example, the prevalence of hypoalbuminemia risk was 12.7% at admission and 45.7% at nadir. Additionally, a previous study showed that the incidence of hypoalbuminemia was approximately 40% in patients receiving CAR-T cell therapy [20]. In a human CD19-targeted mouse model, CAR-T cells could immunologically target and attack cells in the gastrointestinal tract [21]. Frederick L Locke et al. found that patients underwent nausea (58%), decreased appetite (51%), diarrhea (44%), vomiting (34%), constipation (30%), and muscular weakness (16%) in phase 1–2 CAR T cell therapy clinical trials [20]. Another possible explanation is that vascular leakage caused by inflammation during CRS might lead to abnormal distribution of serum albumin, which requires further research.

Numerous studies have highlighted the consequences of malnutrition in cancer patients.

One common consequence of malnutrition is an increased risk of infection, including oral ulcers, perianal infections, lung infections, or outbreaks of Epstein-Barr virus, cytomegalovirus, or hepatitis B virus, which are common in patients with hematological malignancies [22]. Meanwhile, Wie et al. found that 10–20% of cancer deaths could be attributed to malnutrition [23]. Except for a greater risk of mortality, hypoalbuminemia also has an adverse impact on healthcare costs and resources, obviously threatening the quality of life of patients [6, 24, 25]. In addition, as for CAR-T cell therapy, the amplification of cart cells requires energy support, which will affect the curative effect.

It is critical to strengthen albumin monitoring and early nutritional interventions. Nutritional assessment of patients on admission is the primary measure. There have been several validated screening tools available for identifying a malnutrition status or a risk of developing malnutrition, such as the Nutritional Risk Screening 2002 (NRS 2002), the Malnutrition Universal Screening Tool (MUST), and the Mini Nutritional Assessment (MNA) [11]. Patients should select oral nutritional supplements (ONS) or enteral nutrition [22] in the early stages of CAR-T cell treatment with advice from nutritional counselors. For long-term malnutrition or low immunoglobulin, immunoglobulin infusion in the later stages of CAR-T therapy is an essential method on a regular basis. In addition, the combination of rehabilitation medicine and psychological counseling allows stable patients to obtain a better quality of life.

There are some limitations of this study. First, the hematological malignancy types of the included patients were not uniform, which may have affected some of the results. Second, albumin data in some patients were missing. Third, the sample size was relatively small. Further studies with larger sample sizes are needed in the future to elucidate the mechanism of hypoalbuminemia in patients with CRS and to explore the best practices for nutritional support.

## Conclusion

Patients who have undergone CRS have a higher risk of post-CAR-T hypoalbuminemia, the incidence of which increases with the duration and severity of CRS. Therefore, it is critical to pay attention to interventions of early nutrition and management of CRS. It suggests that patients treated with CART should be evaluated early and strengthened with nutritional support, close monitoring during treatment, and multidisciplinary psychophysical support.

### Supplementary Information

**Additional file 1.**

## Data Availability

All analyzed data are included in this published article. The original data are available upon reasonable request to the corresponding author.
