# Supplementary material for: Cytokine release syndrome was an independent risk factor associated with hypoalbuminemia for patients with relapsed/refractory hematological malignancies after CAR-T cell therapy
Source: BMC Cancer. 2023 Nov 2;23:1055. doi: 10.1186/s12885-023-11540-8 (PMC10621126; doi:10.1186/s12885-023-11540-8)
Supplement: Supplementary file 1 — Additional file 1. [file 12885_2023_11540_MOESM1_ESM.docx]

**Supplementary table ASTCT CRS Consensus Grading[1]**

| CRS Parameter | Grade 1 | Grade 2 | Grade 3 | Grade 4 |
| --- | --- | --- | --- | --- |
| Fever* | Temperature≥38°C | Temperature≥38°C | Temperature≥38°C | Temperature≥38°C |
|  | | With | | |
| Hypotension | None | Not requiring vasopressors | Requiring a vasopressor with or without vasopressin | Requiring multiple vasopressors (excluding vasopressin) |
|  | | And/or | | |
| Hypoxia | None | Requiring low-flow nasal cannula or blow-by | Requiring high-flow nasal cannula facemask, nonrebreather mask, or Venturi mask | Requiring positive pressure (eg, CPAP, BiPAP, intubation an |

Organ toxicities associated with CRS may be graded according to CTCAE v5.0 but they do not influence CRS grading.

* Fever is defined as a temperature of 38°C not attributable to any other cause. In patients who have CRS and then receive antipyretic or anti-cytokine therapy such as tocilizumab or steroids, fever is no longer required to grade subsequent CRS severity. In this case, CRS grading is driven by hypotension and/or hypoxia.

CRS grade is determined by the more severe event: hypotension or hypoxia not attributable to any other cause. For example, a patient with a temperature of 39.5° C, hypotension requiring 1 vasopressor, and hypoxia requiring a low-flow nasal cannula is classified as grade 3 CRS.

The low-flow nasal cannula is defined as oxygen delivered at ≤6 L/minute. Low flow also includes blow-by oxygen delivery, sometimes used in pediatrics. The high-flow nasal cannula is defined as oxygen delivered at >6 L/minute.

1. Lee DW, Santomasso BD, Locke FL, Ghobadi A, Turtle CJ, Brudno JN, et al. ASTCT Consensus Grading for Cytokine Release Syndrome and Neurologic Toxicity Associated with Immune Effector Cells. Biol Blood Marrow Transplant. 2019;25(4):625-38.
